# Risk Factors for Death in Patients With Dengue Fever in Dhaka, Bangladesh: A Verbal Autopsy Study

**DOI:** 10.7759/cureus.94380

**Published:** 2025-10-12

**Authors:** Afroza Akter, Imam Tauheed, Md. Golam Firoj, Md. Taufiqur Rahman Bhuiyan, Mohammad Shafiul Alam, Monira Sarmin, Abu Sadat Mohammad Sayeem Bin Shahid, Tahmina Alam, Md. Farhad Kabir, Shahriar Ahmed, Sadia Sabrina, Md. Shahinur Rahaman, Md. Nazmul Islam, Tahmina Shirin, AKM Johirul Hossain Khan, Md. Monjurul Haque, Md. Khairul Islam, Lubaba Shahrin, Mohammed Jobayer Chisti, Sayera Banu, Firdausi Qadri, Fahima Chowdhury

**Affiliations:** 1 Infectious Diseases Division, International Centre for Diarrhoeal Disease Research, Bangladesh (icddr,b), Dhaka, BGD; 2 Clinical and Diagnostic Services, International Centre for Diarrhoeal Disease Research, Bangladesh (icddr,b), Dhaka, BGD; 3 Nutrition Research Division, International Centre for Diarrhoeal Disease Research, Bangladesh (icddr,b), Dhaka, BGD; 4 Communicable Disease Control (CDC), Directorate General of Health Services (DGHS), Dhaka, BGD; 5 Emerging and Remerging Infections, Institute of Epidemiology, Disease Control and Research (IEDCR), Dhaka, BGD; 6 Hospital Administration, Dhaka North City Corporation (DNCC) Hospital, Dhaka, BGD; 7 Department of Medicine, Mugda Medical College and Hospital, Dhaka, BGD; 8 Department of Medicine, Dhaka Medical College and Hospital, Dhaka, BGD

**Keywords:** bangladesh, dengue, respiratory distress, verbal autopsy, vomiting, warning signs

## Abstract

Background

Bangladesh experienced its highest-ever dengue mortality with more than 1,700 deaths in 2023. Despite this high mortality, the data related to causality and circumstances for the fatality of dengue patients in Bangladesh are very limited. The purpose of this study was to identify the clinical characteristics and risk factors related to deaths in dengue patients.

Methods

A verbal autopsy study was conducted between February to November 2024 from three dengue-dedicated hospitals in the Dhaka metropolitan area. Cases were identified from the hospital's registry, following ethical approval from the institutional review board. A total of 82 death cases were recorded between February and November 2024. Written informed consent was obtained from the caregivers of the 17 deceased cases for interviews who lived in Dhaka. We included 17 age-matched severe dengue survivors from these hospitals as the comparison group to identify the risk factors of dengue death.

Results

The median duration from symptom onset to death was five days (interquartile range (IQR): four to eight days) and from hospitalization to death was two days (IQR: one to four days). A total of 10 patients among the 17 (59%) died within the first three days of hospital admission, and the death rate was higher among women (11/17, 64.7%). Respiratory distress was significantly more common in deceased patients compared to survivors (p=0.016). As warning signs, the presence of four or more warning signs (p=0.017), persistent vomiting (p=0.013), and clinical fluid accumulation (p=0.001) were significant indicators of dengue fatality.

Conclusion

The results suggest that the presence of respiratory distress, persistent vomiting, clinical fluid accumulation, and multiple warning signs was found to be associated with dengue fatality. Early identification and prompt management may reduce deaths from dengue.

## Introduction

Dengue is an acute systemic disease having different presentations, from dengue fever (DF), expanded dengue syndrome (EDS), to deadly dengue hemorrhagic fever (DHF) and dengue shock syndrome (DSS) [1]. It is caused by the dengue virus (DENV), which is a member of the genus flavivirus and family Flaviviridae, consisting of four serotypes (DENV-1, -2, -3, and -4) and is transmitted by the mosquito vector, *Aedes aegypti* [2]. The dengue cases reported to the World Health Organization (WHO) have increased intensely, from 0.5 million in 2000 to 14 million in 2024 [3,4]. According to the WHO, dengue is now endemic in more than 100 countries [5], and 40% of the world's population is located in dengue-endemic zones [6]. It has been estimated that 400 million people are affected by dengue, and 21,000 deaths are attributed to dengue annually [7].

In Bangladesh, DENV was first recognized in 1964, though life-threatening DHF started in 2000 [8]. Unplanned rapid urbanization, global warming, heavy rainfall with prolonged rainy seasons, and high humidity have contributed to recent dengue outbreaks in Bangladesh [9,10]. The dengue epidemic in 2023 in urban Dhaka resulted in thousands of deaths and affected 321,179 patients [11]. Previous studies in Bangladesh were related to dengue diagnosis, disease severity, and management. Insufficient data are available on risk factors connected with dengue death, including co-morbidities, which may contribute more to a higher mortality. Studies in Singapore and Pakistan showed that comorbidity was identified as the major cause of death among severe dengue patients [12,13]. There is a lack of locally available guidelines on reporting and managing these risk factors to reduce the death rate among dengue patients.

Verbal autopsy (VA) is an alternative method to collect mortality data to determine the cause of death and circumstances by interviewing the family members or relatives of deceased individuals [14,15]. The VA tool comprises a methodical interview with the caregiver of the death cases to determine the clinical characteristics of the illness that led to the death, as well as a review of available medical records.

This study describes the clinical characteristics and risk factors associated with deaths among dengue patients in Dhaka, Bangladesh, as confirmed by fever for two to six days with either reactive non-structural protein 1 (NS1) or dengue immunoglobulin G (IgG) and IgM.

## Materials and methods

Study sites

**Figure 1:**
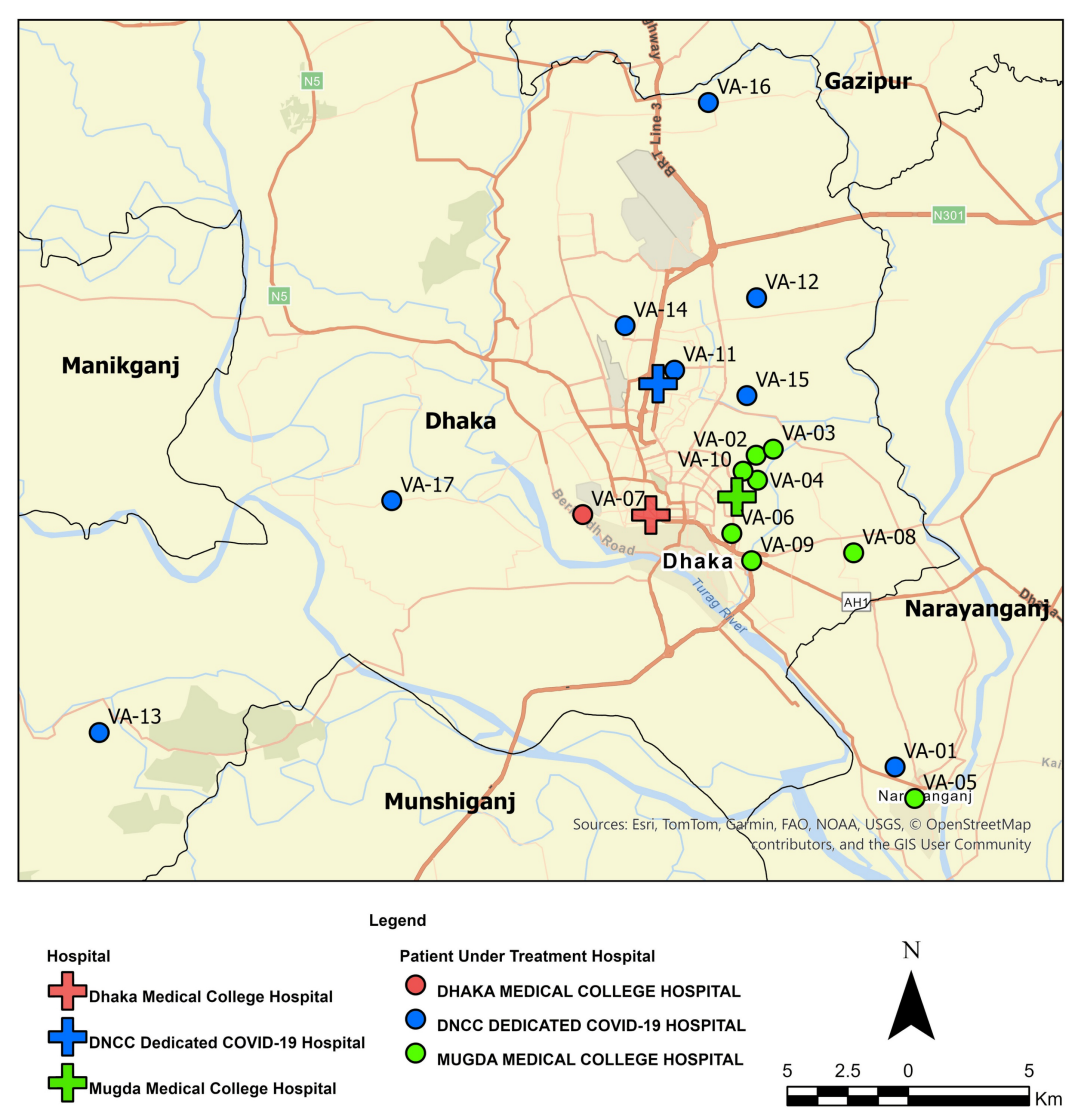
Geographical distribution of deceased dengue cases along with healthcare facilities

This verbal autopsy (VA) study was conducted as a secondary objective of a larger observational study on dengue patients. We conducted an observational cohort study in four hospitals: Dhaka North City Corporation Hospital (DNCC), Mugda Medical College and Hospital (MuMCH), Dhaka Medical College Hospital (DMCH), and International Centre for Diarrhoeal Disease Research, Bangladesh (icddr,b) hospital between December 2023 and January 2025 with the primary objective of identifying the clinical and laboratory factors associated with the progression of severe dengue among hospitalized patients. As part of this larger study, we carried out a nested VA sub-study in three of the hospitals (DNCC, MuMCH, and DMCH) in the Dhaka metropolitan area between February to November 2024 (Figure 1). The objective of this study was to identify the clinical characteristics and risk factors related to deaths in dengue patients.

Verbal autopsy team

All VA interviews were conducted by the designated study physicians in the presence of a study attendant. Before initiation of the study, the interviewer completed specific training on the study-specific VA questionnaire. The training included interview techniques, ethical considerations, and methods to minimize recall bias, ensuring standardized and reliable data collection.

Inclusion process of the participants

Demographic information of the deceased participants was collected from the hospitals to communicate with family members or primary caregivers who were present during the deceased's illness preceding death. We included participants aged ≥18 years old for the VA who resided in and around Dhaka city, and the family members of the deceased participants agreed to be interviewed for this study. We collected the contact details from hospital registries, and the study team attempted to reach each family by phone calls initially. The study objectives, procedures, and benefits were briefly explained over the phone. After verbal consent from the participant, a home visit was scheduled to conduct the interview at the participant’s residence. For each instance where the respondent of a death case did not answer the calls, multiple attempts were made at different times and on different days to establish contact.

We included age-matched severe dengue survivors admitted to the same hospitals and during the same outbreak period. We chose severe survivors as controls to compare them with the deceased to identify risk factors associated with death in dengue patients and factors that may have ultimately contributed to mortality.

Case identification

Between February and November 2024, a total of 82 dengue-related deaths were recorded across three hospitals in Dhaka city (DNCC Hospital, n=28; MuMCH, n=20; and DMCH, n=34). Of these, 43 cases were excluded as they resided outside of Dhaka city, incorrect phone numbers were found for 16 death cases, and six families declined to participate in the study. Consequently, verbal autopsy interviews were completed only for 17 deceased cases.

Ethical consideration

This study was approved by the Research Review Committee and the Ethical Review Committee of icddr,b, and we also obtained approval from the Institutional Review Boards of DNCC, MuMCH, and DMCH. Written informed consent was taken from the participant or participant's guardian and the primary caregiver or family members of the deceased cases before the interview.

Data collection tool

We collected data related to socio-demographics, clinical characteristics, comorbidities, laboratory findings, and treatment of severe dengue cases from the hospitals. For this analysis, we included the socio-demography, clinical characteristics, and co-morbidities data of 17 age-matched, alive severe dengue patients with deceased participants.

In-person interviews of the primary caregivers or family members of deceased participants were conducted for about one to two hours at their homes using a customized VA questionnaire (dengue-specific and country context) developed by the Institute of Epidemiology, Disease Control and Research (IEDCR), Bangladesh, which was adapted from the WHO VA 2022 instrument [16]. We used a fully structured questionnaire with a few open questions administered to conduct the systematic retrospective inquiry of caregivers about the signs and symptoms of illness before death and other associated information to determine the underlying cause and risk factors of death. We visited the households of the deceased participants at least one month after death, and data were collected at a single point to record all information about the deceased patients developed during their illness. The total conversation was audio recorded by the recorder with the interviewer's prior permission.

Abdominal pain, persistent vomiting (more than three times per day), persistent diarrhoea (more than three times per day), clinical fluid accumulation (clinically detectable pleural effusion or ascites), mucosal bleed, lethargy, restlessness, liver enlargement (>2 cm from right costal margin at right mid-clavicular line), and increased hematocrit (HCT) (20%) with decreased platelet count (≤100,000 cell/mm^3^) were considered as the warning signs in this study, which was defined by the Pocket Guideline for Dengue Clinical Case Management of Bangladesh [17,18].

Statistical analysis

The collected data were double-entered into an Excel sheet (Microsoft Office 2013, Microsoft, Redmond, WA) to confirm data accuracy, and analysis was performed using SPSS 20.0 (IBM Corp, Armonk, NY). Demographic, clinical characteristics, warning signs, and comorbidities of survivors and deceased participants were stratified. We measured the percentage for categorical data and median with inter-quartile range (IQR) for ordinal data. Associations between patient characteristics and survival status (survived vs. deceased) were examined using the chi-square test. For variables with sparse data, Fisher’s exact test was applied to ensure statistical validity, and p<0.05 was considered statistically significant.

## Results

**Figure 2:**
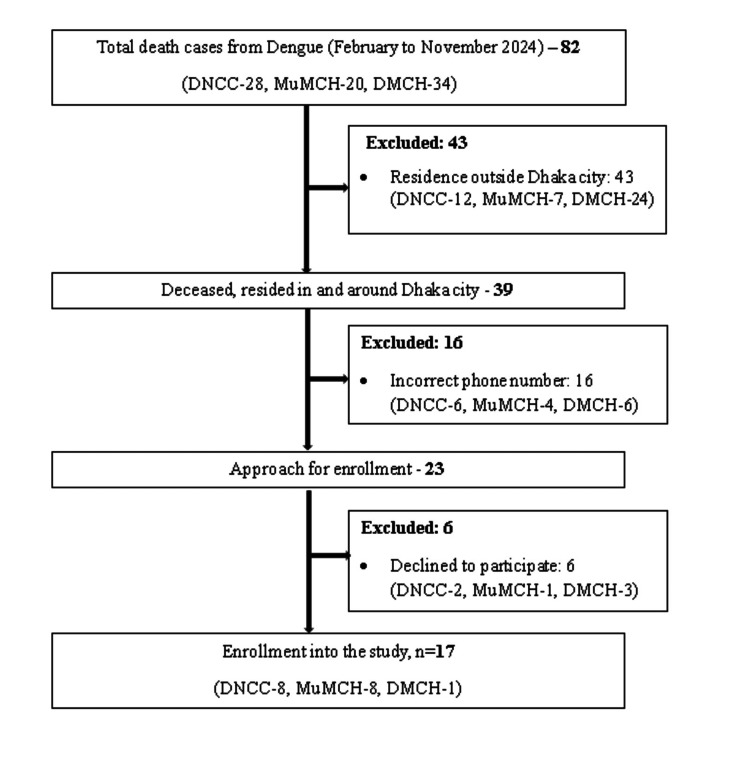
Participant enrollment flow diagram

A total of 82 dengue death records were found from DNCC (28), MuMCH (20), and DMCH (34) from February 2024 to November 2024. Upon fulfilling the inclusion criteria, we completed 17 VA interviews from March 2024 to January 2025: eight interviews from DNCC, eight from MuMCH, and one from DMCH (Figure 2). 

We found a total of 62 severe dengue cases from DNCC, MuMCH, DMCH, and icddr,b hospitals from the primary study. From these 62 cases, 17 age-matched and alive severe dengue patients were included in the study: eight from DNCC, seven from MuMCH, one from DMCH, and one from icddr,b Dhaka hospital for comparison.

The median age among survivors was 45 years (IQR: 32, 56) compared to 49 years (IQR: 28, 54) among the deceased, showing no statistically significant difference. The distribution of gender and education level among both the survivors and deceased groups was similar (Table 1).

**Table 1:** Socio-demographic characteristics of dengue cases *Fisher’s exact test. †Chi-square test. p<0.05 was considered statistically significant.

Variables	No. (%) of survivor cases (n=17)	No. (%) of death cases (n=17)	p-values*
				Age			
<18	2 (11.8%)	2 (11.8%)	>0.999
18-45	6 (35.3%)	6 (35.3%)	
≥45	9 (52.9%)	9 (52.9%)	
Median age (interquartile range, IQR)	45 (32, 56)	49 (28, 54)	
Gender			
Female	10 (58.8%)	11 (64.7%)	>0.999^†^
Male	7 (41.2%)	6 (35.3%)	
Level of schooling			
No formal education (0 years of schooling)	2 (11.8%)	2 (11.8%)	0.832
Primary (1-5 years schooling)	7 (41.2%)	7 (41.2%)	
Secondary (6-10 years schooling)	4 (23.5%)	6 (35.3%)	
Higher (≥11 years of schooling)	4 (23.5%)	2 (11.8%)	

**Figure 3:**
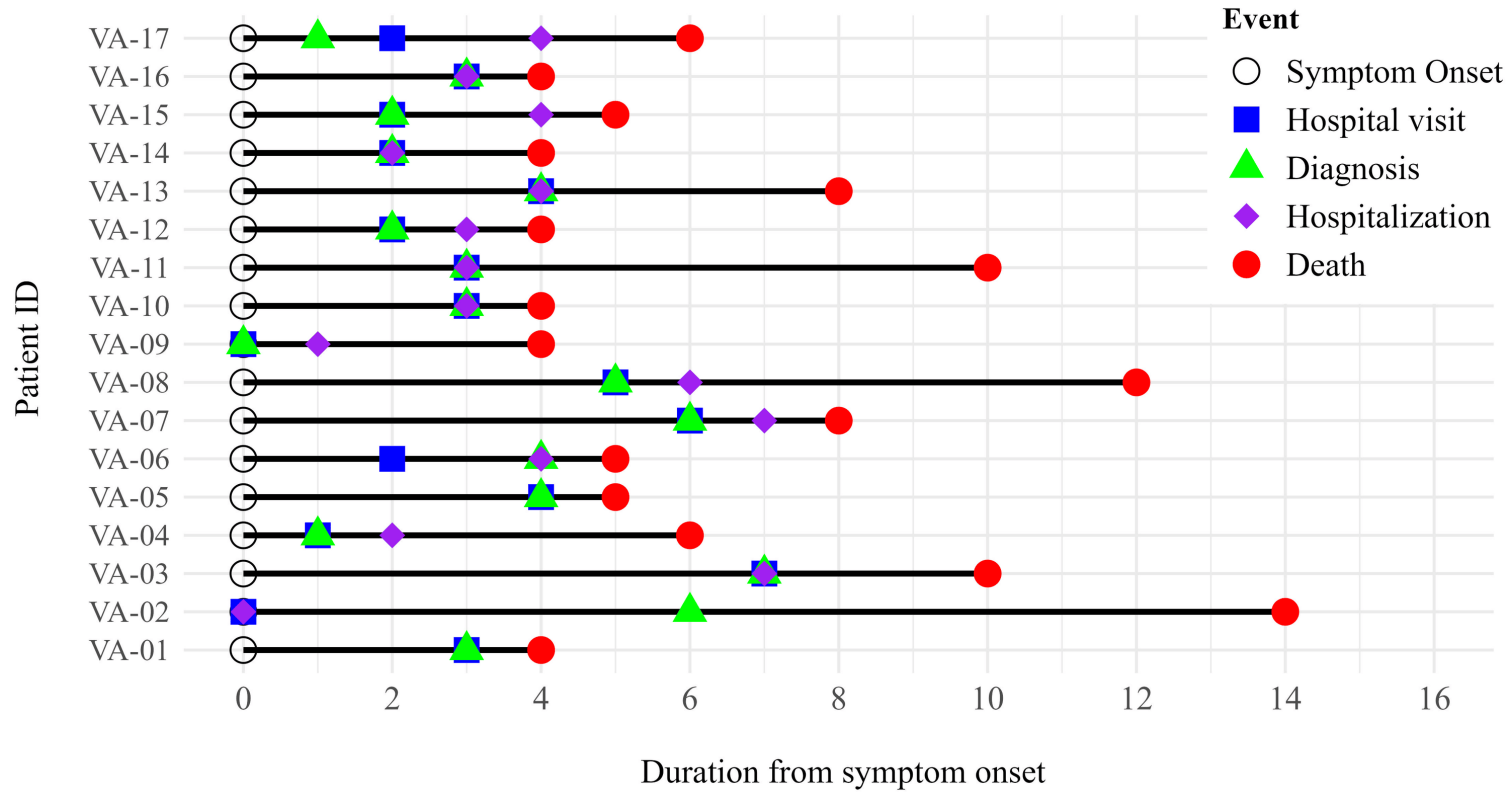
Events of the dengue death cases Seventeen death cases were individually represented, showing the events starting from symptom onset till death in days (X-axis). The Y-axis specified the date of the first symptom onset.

The median duration from symptom onset to hospitalization was four days in both the survivor and deceased groups. In the survivor group, the median total hospital stay was six days (range, two to 14 days), and the median total duration from symptom onset to disease resolution was 10 days (range, five to 21 days). The median duration from symptom onset to death was five days (range, four to 14 days), and the median time from positive dengue test to death was three days (range, one to eight days). The median duration from hospitalization to death was two days (range, 0-14 days). The majority of patients (10/17, 59%) died within the first three days of hospital admission (Figure 3).

All participants in both survivor and death groups presented with fever. Vomiting was more frequent among the deceased (11/17, 64.7%) compared to survivors (7/11, 41.2%), though the difference was not statistically significant (p=0.302). Respiratory distress or breathlessness was significantly more common in deceased patients (8/11, 47.1%) compared to survivors (1/17, 5.9%) (p=0.016), highlighting its association with poor outcomes. A striking difference was observed in the occurrence of headache, which was significantly more common in survivors (15/17, 88.2%) compared to deceased (2/17, 11.8%) (p<0.001). Other clinical symptoms, such as body ache/back pain, bleeding (gum, nasal, rectal, urethral, or conjunctival), abdominal pain or distension, restlessness, fatigue, confusion, cough, chest pain, fall/sudden fall, rash, and vertigo, presented no statistically significant differences between the survivor and death groups (Table 2).

**Table 2:** Comparison of clinical features by hospital outcome (survivor or death) *Fisher’s exact test. ^†^Chi-square test. p<0.05 was considered statistically significant.

Clinical Feature	No. (%) of survivor cases (n=17)	No. (%) of death cases (n=17)	p-values*
				Fever	17 (100)	17 (100)	>0.999
Vomiting	7 (41.2)	11 (64.7)	0.302^†^
Body ache/back pain	11 (64.7)	10 (58.8)	>0.999^†^
Respiratory distress/breathlessness	1 (5.9)	8 (47.1)	0.017
Bleeding (gum, nasal, rectal, urethral, conjunctival)	4 (23.5)	6 (35.3)	0.707
Abdominal pain/distension	11 (64.7)	6 (35.3)	0.170^†^
Headache	15 (88.2)	2 (11.8)	<0.001
Restlessness/lethargy	7 (41.2)	3 (17.65)	0.259
Fatigue	16 (94.1)	17 (100)	>0.999
Confusion	0	2 (11.8)	0.484
Cough	7 (41.2)	2 (11.8)	0.117
Chest pain	0	2 (11.8)	0.484
Fall/sudden fall	0	2 (11.8)	0.484
Rash	3 (17.65)	1 (5.9)	0.601
Vertigo	0	1 (5.9)	>0.999

All survivor cases had at least one warning sign during their disease course, and all death cases had two or more warning signs. Among the death cases, eight (47.1%) had four or more dengue warning signs. In contrast, only one (5.9%) of the survivors had four or more warning signs. The association between the presence of four or more warning signs and mortality was statistically significant (p=0.017), indicating that a higher number of warning signs was significantly associated with fatal outcomes. The presence of specific clinical warning signs differed notably between survivors and deceased dengue patients. Persistent vomiting was significantly more common among those who died compared to survivors (Table 3). Clinical fluid accumulation was observed exclusively among deceased patients, with none of the survivors presenting this feature, indicating a strong association with mortality (Table 3). Other warning signs, such as mucosal bleeding, increased hematocrit with decreased platelet count, abdominal pain, lethargy, and liver enlargement, were seen in both groups without statistically significant differences. Interestingly, persistent diarrhoea was reported only in survivors (4/17, 23.5%), but this inverse relationship with mortality was not statistically significant (p = 0.103) (Table 3).

**Table 3:** Distribution of warning signs among the dengue cases stratified by hospital outcome HCT: Hematocrit. *Fisher’s exact test. ^†^Chi-square test. p<0.05 was considered statistically significant.

Warning signs	No. (%) of survivor cases (n=17)	No. (%) of death cases (n=17)	p-values*
				Patients with four or more warning signs	1 (5.9)	8 (47.1)	0.017
Persistent vomiting	3 (17.65)	11 (64.7)	0.013
Increased HCT with decreased platelets	15 (88.2)	11 (64.7)	0.225
Clinical fluid accumulation	0	9 (52.9)	0.001
Mucosal bleeding	4 (23.5)	8 (47.1)	0.281
Abdominal pain	7 (41.2)	6 (35.3)	>0.999^†^
Restlessness/lethargy	7 (41.2)	3 (17.65)	0.259
Liver enlargement >2 cm	0	1 (5.9)	>0.999
Persistent diarrhoea	4 (23.5)	0	0.102

Among the death cases, 10 (58.8%) had at least one co-morbidity, with hypertension in seven, diabetes mellitus in six, hypothyroidism in two, cardiovascular diseases in one, kidney disease in one, and chronic lung diseases in one. Five deceased cases had more than one comorbidity. No statistically significant differences were observed between survivor and death groups for the presence of any individual co-morbidity or multiple co-morbidities (Table 4).

**Table 4:** Co-morbidities among the dengue cases *Fisher’s exact test. ^†^Chi-square test. p<0.05 was considered statistically significant.

Variables	Survivor cases (n=17)	Death cases (n=17)	p-values*
Patients with comorbidities	9	10	>0.999^†^
Patients with multiple comorbidities	2	5	0.398
Hypertension	3	7	0.258
Diabetes mellitus	4	6	0.707
Hypothyroidism	0	2	0.484
Cardiovascular diseases	0	1	>0.999
Kidney disease	0	1	>0.999
Chronic lung diseases	2	1	>0.999

## Discussion

Identifying dengue cases according to the severity grading is important for appropriate management. This is the first study in Bangladesh to investigate the clinical characteristics and risk factors associated with the fatality of dengue cases, which aligns well with our study objectives. The use of VA provided a unique insight and better understanding into disease progression and the socio-clinical contexts preceding death, filling a significant gap in Bangladesh’s mortality surveillance related to dengue.

Our data showed that all 17 death cases presented with fever and fatigue and were admitted to the hospital with two or more warning signs. One of our key observations was that most deaths occurred within the first three days of hospital admission, underscoring the narrow window for effective clinical intervention. In comparison, the median time from symptom onset to hospitalization was similar in both survivor and deceased groups (four days) to death.

We found a significant association between the number of dengue warning signs and dengue-related death. Specifically, the presence of four or more warning signs and dengue mortality was statistically significant, which indicates that a higher number of warning signs was significantly associated with fatal outcomes. A similar observation was reported in a study of Malaysia, where dengue patients who died often had four or more warning signs [19]. Our results suggest that the cumulative presence of multiple warning signs may serve as a critical early indicator of deterioration in patients with dengue. Timely intervention in such high-risk cases may help reduce dengue-related mortality.

We found that respiratory distress or breathlessness was a significant common clinical feature in deceased patients compared to survivors, and this finding was similar to a study conducted in Kerala, India [20]. Similarly, in another study in Colombia, a significantly higher death rate was observed among the dengue patients with respiratory distress independently [21]. In contrast, our study revealed that headache was significantly prevalent among survivors despite being one of the common symptoms of severe dengue. This finding suggests that the caregivers of the deceased cases inadequately described the headaches. Our findings highlight the prognostic significance of warning signs such as persistent vomiting and clinical fluid accumulation of fatal outcomes in dengue. Similar patterns have been reported in another dengue-endemic country, Sri Lanka, where fluid leakage and persistent vomiting were noted as indicators of disease severity [22]. This symptom likely reflects underlying plasma leakage, which can lead to pleural effusion, fluid accumulation, and eventually respiratory compromise.

A high prevalence of comorbid conditions, particularly hypertension and diabetes, was observed in both survivors and deceased, although these were not statistically significant. This highlights a potential role of chronic disease management in dengue outcomes. Prior studies in Singapore and Pakistan have underscored the synergistic effect of comorbidities in worsening clinical trajectories among dengue patients [12,13]. Integrating comorbidity screening into dengue case management could refine risk stratification.

In our study, we found that the median duration from symptom onset to hospital admission was four days in the deceased group, which is very similar to the study by Kandel et al. (2025), which shows that around half of the fatal cases were hospitalized within five days of the onset of symptoms [23]. Of particular concern is the median time from hospitalization to death being only two days, with over 50% dying within the first 72 hours of hospital admission. This rapid progression to death supports prior reports from Nepal, which noted that 46.8% patients died within three days of hospitalization [23]. Strengthening early warning systems, community-based education, and primary care recognition of critical symptoms may be crucial in reducing mortality due to dengue.

This study also highlights the utility of VA tools in urban dengue surveillance. Furthermore, public health planning must account for the compressed timeframe between diagnosis and death. This implies a need for protocolized triage systems, especially during outbreak peaks when healthcare systems are overwhelmed. Predictive analytics and decision support tools, which utilize warning signs and comorbidity profiles, can aid in real-time clinical decision-making. A retrospective study in Singapore demonstrated that predictive tools using clinical warning signs and demographic factors can enhance triage for severe dengue, reducing unnecessary admissions by 19% and supporting real-time clinical decision-making during outbreaks [24].

This study has several limitations. The study included only 17 interviews, which limits statistical power and reduces the generalizability of our findings. Non-inclusion of other deaths was largely due to residence outside Dhaka, incorrect contact information, or refusal. Although interviews were conducted by a clinically trained physician using a standardized tool, recall bias and selection bias remain possible. The VA method also lacks real-time clinical observation and postmortem confirmation, which may have led to underreporting of subtle signs such as bleeding or hepatomegaly. Furthermore, we did not assess laboratory parameters like HCT trends or viral serotype distributions in the deceased cases, which could further clarify pathophysiological mechanisms. Finally, this was a hospital-based study in a highly urbanized setting, and findings may not reflect dengue mortality patterns in rural or less resourced settings. Future studies should address these limitations by using larger, multicentre, and prospective designs with broader inclusion criteria and improved follow-up strategies to strengthen representativeness.

## Conclusions

This study provides important preliminary evidence on risk factors associated with dengue mortality in Bangladesh during its largest outbreak to date. The findings highlight respiratory distress, persistent vomiting, clinical fluid accumulation, and multiple warning signs as key predictors of fatality. We recommend that hospital triage protocols prioritize these high-risk indicators to ensure timely recognition and prompt management of severe cases. Vaccination, improved diagnostic tools, and clinical management of dengue patients can help reduce dengue mortality. Policies should be dynamic to adapt to these changing patterns of dengue transmission and mortality, and need special consideration of urbanization trends and climate change impacts.
